# Catastrophic Antiphospholipid Syndrome Presenting as a Stroke in an 11-Year-Old with Lupus

**DOI:** 10.1155/2022/7890566

**Published:** 2022-05-13

**Authors:** Brooke Senken, Anne Whitehead

**Affiliations:** ^1^Riley Hospital for Children, Indianapolis, IN, USA; ^2^Department of Emergency Medicine, Indiana University School of Medicine, Indianapolis, IN, USA

## Abstract

Catastrophic antiphospholipid syndrome (CAPS) is an infrequent but feared life-threatening complication of antiphospholipid syndrome (APS). CAPS is characterized by the rapid development of numerous thromboses across multiple organs resulting in multiorgan failure. It is rare but well-documented in the adult population. In contrast, it is exceedingly uncommon in pediatric patients and therefore not yet well described in the pediatric literature. Early recognition of APS is of the utmost importance to provide timely and effective management for a positive outcome. We present the case of an 11-year-old girl with history of systemic lupus erythematosus (SLE) and hypertension (HTN) who presented with acute onset altered mental status, found to have a large ischemic middle cerebral artery (MCA) and anterior cerebral artery (ACA) stroke as well as multiple, diffuse, and smaller ischemic lesions in the frontal lobe and cerebellum. Her presentation was further complicated by thrombocytopenia and renal and splenic infarction, as well as thrombosis of the right brachial vein consistent with a diagnosis of CAPS.

## 1. Introduction

Antiphospholipid syndrome (APS) is an autoimmune disorder defined by recurrent arterial and venous thrombosis associated with elevated titers of antiphospholipid antibodies including lupus anticoagulant, anti-*β*2-glycoprotein, and anti-cardiolipin antibodies [1]. In the pediatric population, the most common clinical presentation is venous thrombosis of the lower extremities, followed by cerebral venous thrombosis and ischemic stroke [2, 3]. A greatly feared and life-threatening complication of APS is catastrophic antiphospholipid syndrome (CAPS), defined as the rapid progression of microvascular thrombosis with multiorgan involvement and subsequent multiorgan failure [4]. CAPS presents in less than 1% of all patients with APS and is even rarer in the pediatric patients with only a handful of cases reported in the literature [5, 6]. Prognosis is poor for children with APS, with estimates of 33% mortality or higher [4]. Most pediatric cases of CAPS are triggered by infections yet can also be precipitated by neoplasms, recent surgery, and SLE flares [2, 4].

The general diagnostic criterion for CAPS requires that at least three organ systems be involved. Common systems comprise of the central nervous system (ischemic stroke, sinus venous thrombosis), pulmonary (pulmonary embolism, acute respiratory distress syndrome, cardiac (myocardial infarction, heart failure), renal (proteinuria, hypertension), skin (cutaneous necrosis, digital ischemia), and solid organs (splenic, renal, and/or adrenal infarct). Important laboratory findings include presence of antiphospholipid antibodies, thrombocytopenia, microangiopathic hemolytic anemia, disseminated intravascular coagulation, and elevated ferritin [2, 4]. Furthermore, CAPS associated with the presence of SLE is more likely to cause severe cardiac and brain involvement and therefore greater mortality [5].

There are no current diagnostic criteria for pediatric CAPS, and therefore, its treatment often follows guidelines published in the adult literature [1, 3, 7]. Anticoagulation and steroids are encouraged for the primary treatment approach. In some cases, therapeutic plasma exchange and intravenous immunoglobulin administration have also been used. More recently, studies have looked at the use of rituximab as an effective pharmacologic agent [4, 7]. The international CAPS registry is a great resource to aide with the understanding of the disease, but data are largely derived from the adult population. Presently, there is no separate registry that focuses on the pediatric population as it relates to the causes, mechanisms, history, and treatment of children affected by CAPS likely because the prevalence is extremely low.

The potentially lethal outcome of CAPS highlights the importance of quick recognition and intervention. Since CAPS is so rare in children, it does not readily come to the forefront of the clinical differential processes and can easily be confused with other pathology leading to delayed diagnosis. We report the case of an 11-year-old female who presented to a pediatric emergency department with a large ischemic stroke secondary as the initial manifestation of CAPS.

## 2. Case Report

**Figure 1:**
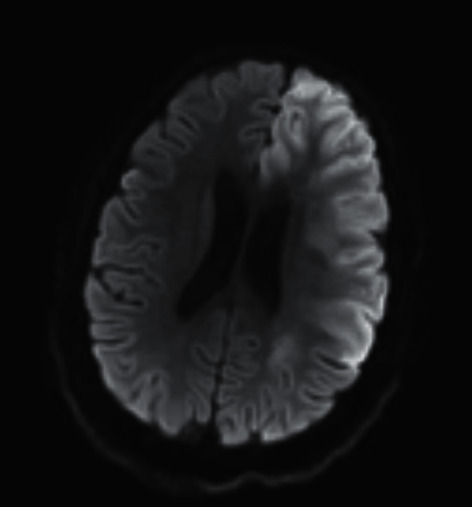
MRI brain without contrast performed during the patient's acute presentation to the emergency department showing large territory left hemispheric stroke.

An 11-year-old girl presented to a pediatric emergency department (ED) via ambulance with altered mental status. Her mother reported finding her unresponsive on the bathroom floor laying in a pool of vomit and diarrhea. She had been in her normal state just minutes prior to being found by her mother, approximately 45 minutes prior to her arrival to the ED. Her mother reported no apparent seizure activity and denied that she had a history of seizures, recent infection, or recent trauma. Her mother reported she had very low suspicion that the patient had ingested any medications or recreational drugs. Her past medical history included HTN and SLE. Her initial vital signs were normal apart from an elevated blood pressure of 135/87 (99^th^ percentile for age). Initial Glasgow Coma Scale (GCS) was 9. She had spontaneous eye opening, no verbal response to stimuli, and did withdraw from painful stimuli. She had right upper extremity moderate hypertonicity and seemed to spontaneously move the left side of her body more than her right. She had an intact gag reflex and no apparent impending airway compromise, so she was not immediately intubated. Initial labs were notable for thrombocytopenia with a platelet count of 11 k/cumm, anemia with hemoglobin of 7.1 g/dl and hematocrit of 21.2%, and an elevated creatinine of 0.83 mg/dl. Moderate schistocytes were seen on peripheral smear. Noncontrasted computed tomography (CT) head imaging was obtained shortly after her arrival to the ED and revealed no acute findings. The case was discussed with the on-call neurologist, and both magnetic resonance imaging (MRI) and CT angiography were considered, and ultimately, given the ready availability of MRI at our hospital at the time of the patient's presentation, and its diagnostic utility for many of the diagnoses high on our differential (which included posterior reversible encephalopathy and lupus cerebritis as well as acute ischemic stroke), we decided to proceed with MRI next. This revealed a large infarct in the left cerebral hemisphere involving the frontal lobe and basal ganglia as well as a few small and acute infarctions in the right frontal lobe and subacute cortical infarctions in the cerebellum (Figure 1).

Prior to this acute event, patient was known to have SLE with cutaneous disease (discoid like rash), oral ulcers, arthritis, proteinuria, hematuria, and positive antiphospholipid antibodies (elevated *β*2-glycoprotein, cardiolipin, and lupus anticoagulant). She had positive antinuclear antibody and positive double-stranded DNA. She had no prior history of cerebrovascular events. Her SLE was stable with use of hydroxychloroquine and prednisolone.

Treatment with systemic tissue plasminogen activator (tPA) was considered, but ultimately not performed as patient had a very high risk of hemorrhagic conversion considering her significant thrombocytopenia and very high NIH Stroke Scale (NIHSS) of 27. Mechanical thrombectomy was also considered, and she was transferred briefly to the adult stroke center less than 1 mile from our pediatric hospital for CT perfusion imaging and evaluation by neurointerventional radiology. Ultimately, she did not undergo thrombectomy as it was contraindicated by her very large ischemic penumbra, and she was transferred back to the pediatric intensive care unit (PICU).

Further imaging and laboratory evaluation was performed once the patient arrived in the PICU. A venous ultrasound showed a thrombus in the right brachial vein. CT abdomen and pelvis revealed multiple cortical infarcts in bilateral kidneys as well as embolic splenic infarcts. Rheumatologic labs revealed presence and elevation of antiphospholipid antibodies including anticardiolipin, anti-*β*2-glycoprotein, and lupus anticoagulant. Concern for microvascular myocardial involvement arose after patient was noted to have elevated troponin and increasing premature ventricular contractions. All these findings were consistent with a diagnosis of CAPS. She required a prolonged PICU stay with multispecialty consultation. Her treatment did ultimately involve systemic anticoagulation with heparin despite her thrombocytopenia and high risk of hemorrhagic conversion given the high mortality associated with ongoing thrombosis in CAPS. Her treatment also included cyclophosphamide, steroids, and plasmapheresis.

She was then transferred to a rehabilitation center due to resulting impairments including right-sided hemiplegia, mixed aphasia, dysphagia, and reduced strength and balance. At her most recent outpatient visit with neurology, roughly one year after her acute presentation, she continued to demonstrate moderate right-sided motor deficits and mild aphasia requiring continued outpatient physical therapy.

## 3. Discussion

CAPS is an extremely uncommon, yet greatly feared variant of APS in the pediatric population. It is a rapidly progressive form of APS consisting of numerous microvascular thrombi leading to multiorgan failure. Mortality is high even in patients with prompt recognition and treatment. Due to its rarity in the pediatric population, little information is known regarding its recognition, natural progression, treatment, and quality of life of the children affected by this disease. Very few papers have been published on CAPS in the pediatric population, and only one other case report describing ischemic stroke as the presenting manifestation of pediatric CAPS. This case described CAPS in the setting of acute myelogenous leukemia rather than SLE [8].

The diagnosis of CAPS was not immediately apparent during our patient's ED presentation. We believe this was in part due to the emergency medical team's lack of familiarity with the diagnosis, and the rarity of this entity in the general as well as pediatric population. It is crucial that emergency and pediatric emergency physicians be aware of this potential diagnosis, as it is important to ensure that patients with suspected CAPS be admitted to centers with the availability of multispecialty teams capable of managing this challenging and rare syndrome.

Our patient presented with an acute arterial stroke. Even in the absence of underlying CAPS, this is an uncommon entity in the pediatric population, with roughly 1000 yearly cases of pediatric stroke in the United States and an estimated incidence of 1.6/100,000 [9, 10]. Emergent pediatric stroke evaluation and treatment has largely depended on findings from the extensive adult literature and case series. Attempts to better study pediatric stroke management, for example, the TIPS study, has been challenged with poor enrollment secondary to the rarity of presentation [11].

Patients with juvenile SLE are at risk for greater disease severity at presentation and higher acuity at presentation as well as more considerable organ involvement and damage when compared with patients with adult-onset SLE. Juvenile SLE with associated nephritis, thrombocytopenia, anti-double stranded DNA, and anti-cardiolipin antibodies places patients at risk for severe disease activity and a overall worse prognosis [12, 13]. Our patient unfortunately had many of these high risk factors which ultimately contributed to her severe and critical ED presentation.

This case highlights the importance of the development and use of pediatric stroke protocols in emergency departments. There were no pediatric-specific stroke imaging or treatment protocols in place in our hospital at the time of our patient's emergency department presentation. As a result, medical decision-making and care coordination were less streamlined and lead to potentially longer times to imaging needed for clinical decision-making which could potentially have impacted outcomes. While our patient ultimately was not considered a good candidate for systemic thrombolytics nor thrombectomy given her high risk for hemorrhagic conversion, this case, and others like it, has led to the development of a pediatric stroke protocol in our hospital system.

We conclude that while CAPS is a rare entity in the pediatric population, it is important that pediatric emergency physicians be aware of this diagnosis and its potential implications. Furthermore, we highlight the need for a more standardized pediatric stroke protocol in the emergency department to help expedite its recognition and prompt treatment. We stress the importance to consider APS in any child who presents with cerebral ischemia and multiorgan involvement, particularly children who have a history of SLE.

## Abbreviations

APS: Antiphospholipid syndrome
ACA: Anterior cerebral artery
CAPS: Catastrophic antiphospholipid syndrome
CT: Computed tomography
Cr: Creatinine
ED: Emergency department
HTN: Hypertension
MCA: Middle cerebral artery
PICU: Pediatric intensive care unit
SLE: Systemic lupus erythematosus.

## Additional Points

An 11-year-old with lupus presenting to the emergency department with acute stroke found to be a result of catastrophic antiphospholipid syndrome.
